# Supplementary material for: Widespread introgression and a potential role for a neo-sex chromosome in booby diversification and speciation
Source: bioRxiv. 2026 Jul 22:2026.07.17.739190. Preprint. [Version 1] doi: 10.64898/2026.07.17.739190 (PMC13419459; doi:10.64898/2026.07.17.739190)
Supplement: Supplementary file 1 [file NIHPP2026.07.17.739190v1-supplement-1.pdf]

bioRxiv preprint doi: https://doi.org/10.64898/2026.07.17.739190; this version posted July 22, 2026. The copyright holder for this preprint (which was not certified by peer review) is the author/funder. This article is a US Government work. It is not subject to copyright under 17 USC 105 and is also made available for use under a CC0 license.

Extended Data Figures

Figure S1. Phylogenetic trees constructed from each chromosome. Chromosome 29 was dropped from the final consensus tree after it was identified as a neo-sex chromosome. Species level monophyly was recovered across analyses of all macrochromosomes except for the relationship between brown and Cocos boobies, for which our analyses recover a deeper split in the brown-Cocos clade between ocean basins than between species.

bioRxiv preprint doi: https://doi.org/10.64898/2026.07.17.739190; this version posted July 22, 2026. The copyright holder for this preprint (which was not certified by peer review) is the author/funder. This article is a US Government work. It is not subject to copyright under 17 USC 105 and is also made available for use under a CC0 license.

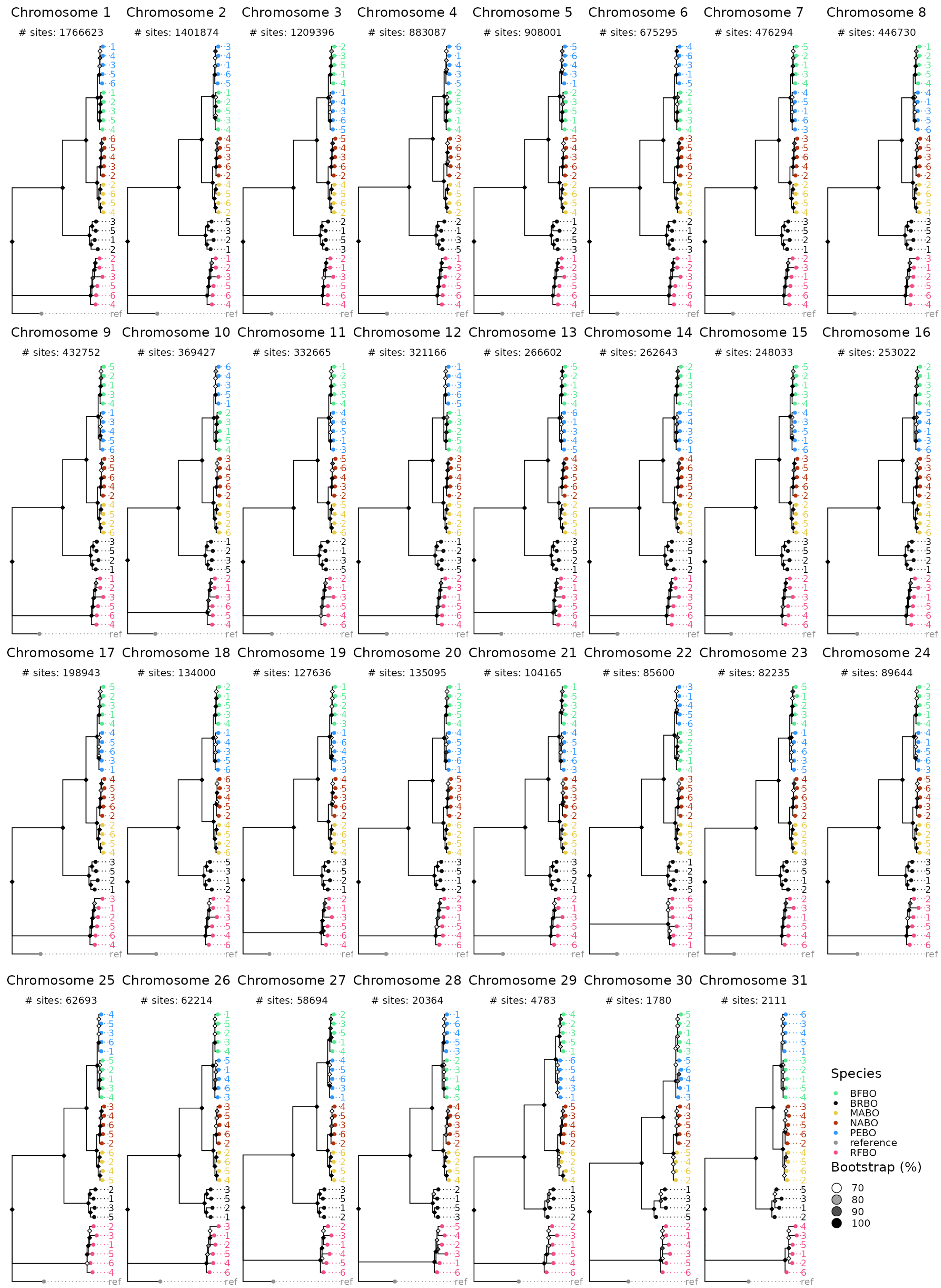

bioRxiv preprint doi: https://doi.org/10.64898/2026.07.17.739190; this version posted July 22, 2026. The copyright holder for this preprint (which was not certified by peer review) is the author/funder. This article is a US Government work. It is not subject to copyright under 17 USC 105 and is also made available for use under a CC0 license.

Figure S2. Results of principal component and ADMIXTURE analyses of all species and of species pairs. The ellipses were manually added to denote sampling locations of clusters that arise from specific ocean basins. Sample sizes are small for within-species analyses, especially for ADMIXTURE analyses, and we therefore weight insights from PCAs over those from ADMIXTURE.

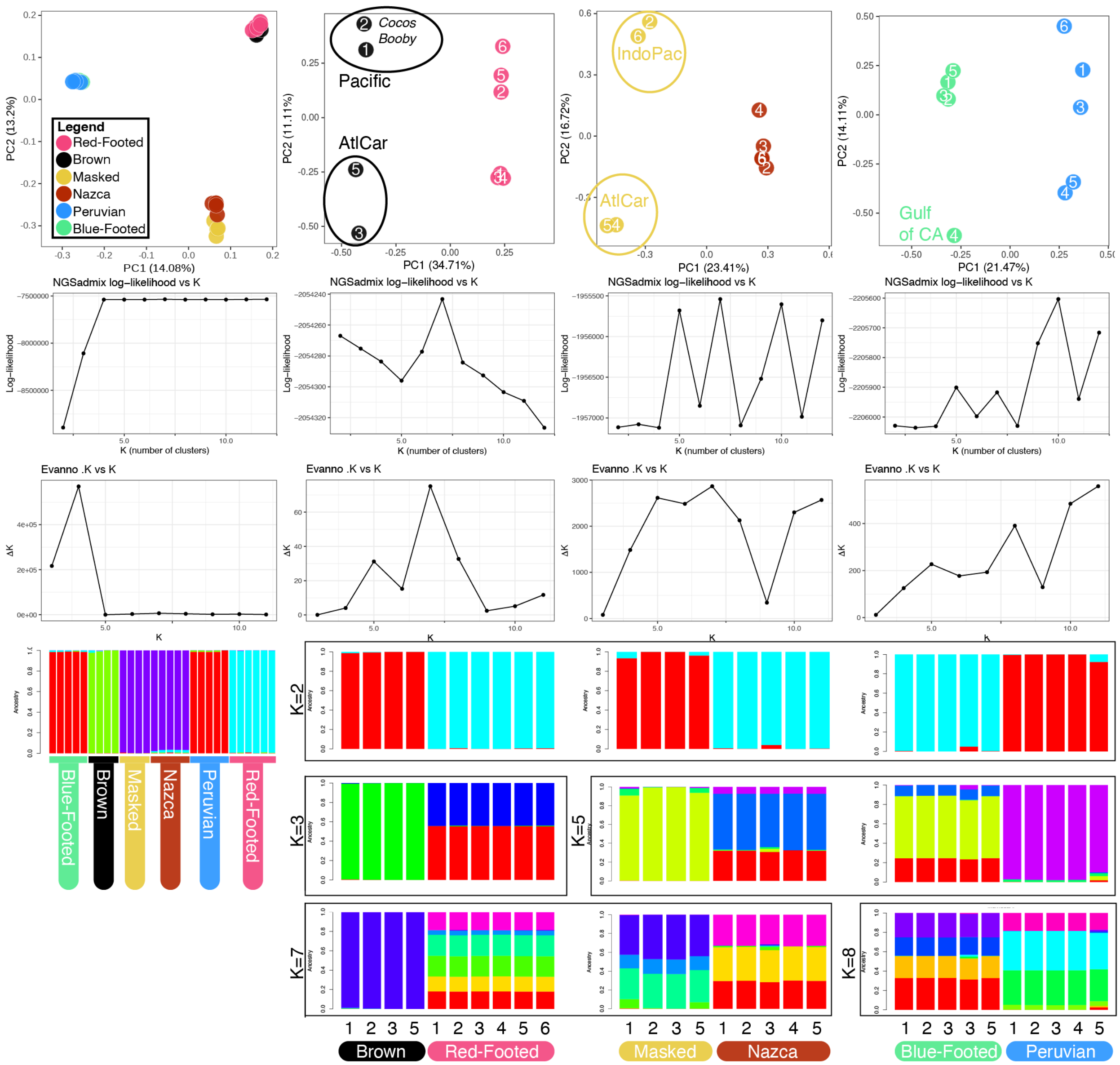

bioRxiv preprint doi: https://doi.org/10.64898/2026.07.17.739190; this version posted July 22, 2026. The copyright holder for this preprint (which was not certified by peer review) is the author/funder. This article is a US Government work. It is not subject to copyright under 17 USC 105 and is also made available for use under a CC0 license.

Figure S3. Results of principal component and ADMIXTURE analyses of populations. The ellipses were manually added to denote sampling locations of clusters that arise from specific ocean basins. Sample sizes are small for within-species analyses, especially for ADMIXTURE analyses, and we therefore weight insights from PCAs over those from ADMIXTURE.

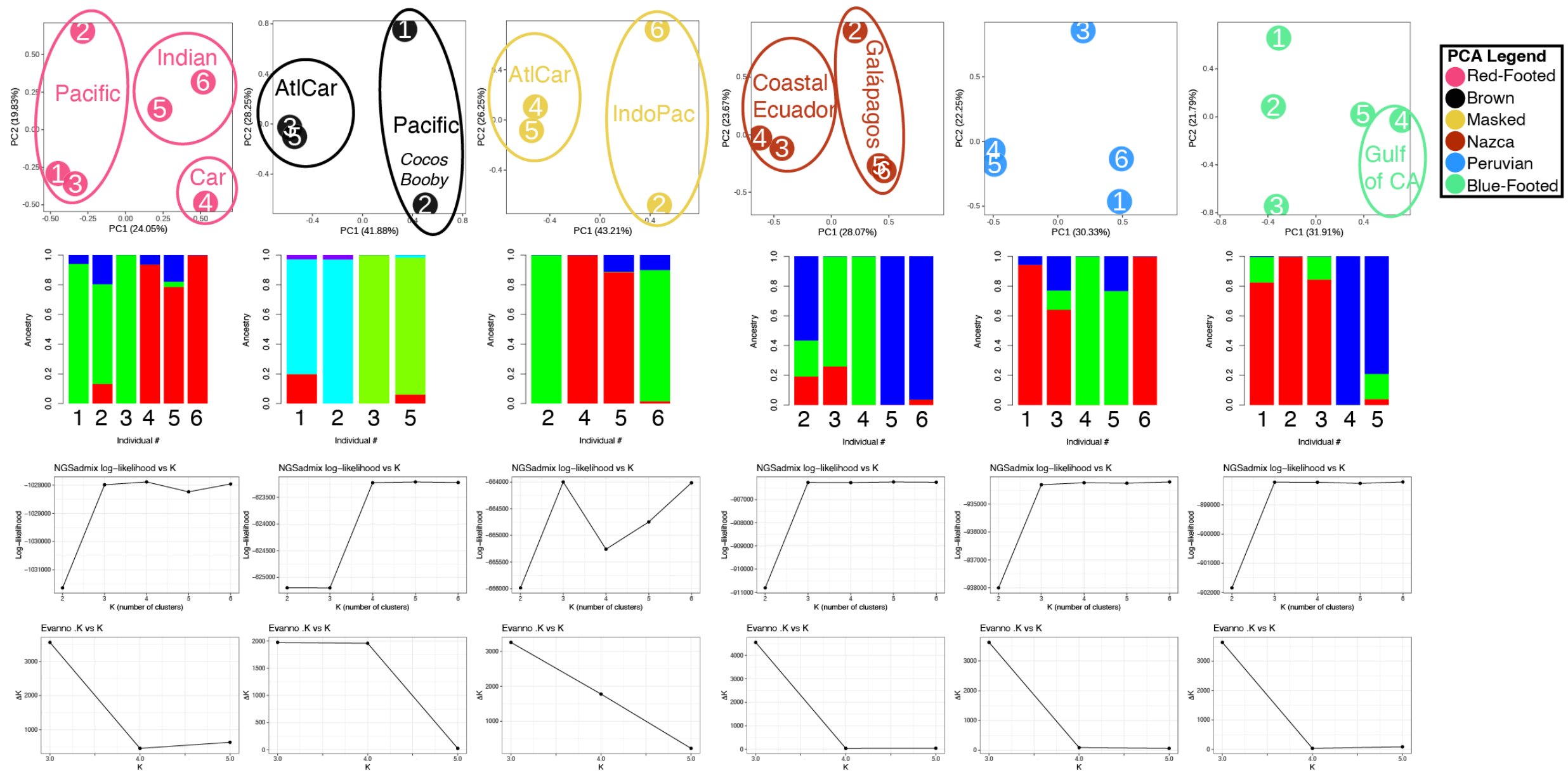

bioRxiv preprint doi: https://doi.org/10.64898/2026.07.17.739190; this version posted July 22, 2026. The copyright holder for this preprint (which was not certified by peer review) is the author/funder. This article is a US Government work. It is not subject to copyright under 17 USC 105 and is also made available for use under a CC0 license.

Figure S4. Demographic history models by individual in MSMC. Plots show the lower and upper bound generation times and are plotted colored by species (top two plots) and by individual (lower six plots). The color of the lines in the "All individuals" plots correspond to the box around each species. Historical demographic modeling of individual genomes was consistent within taxa except for red-footed and brown-Cocos boobies (Figure S6). All species showed a sharply declining trend prior to one million years ago, and masked, Nazca, Peruvian, and blue-footed boobies remained at that low effective population size over time. Genomes from different populations of red-footed boobies varied over the last million years, with the samples from the eastern Pacific and Caribbean declining in recent years and all other samples increasing. The central Pacific brown booby sample is an outlier from other brown booby samples, and this genome demonstrated population size expansion over the last million years, while other brown booby samples had patterns of genetic variation consistent with declining effective population size. The Cocos booby, central Pacific masked booby samples, and equatorial blue-footed booby samples showed a slightly higher effective population size compared to their conspecifics (or for the Cocos booby, compared to brown boobies) around 10,000–100,000 years ago. All Peruvian booby samples increased in effective population around that time as well. The Gulf of California blue-footed booby sample demonstrated a lower effective population size than the equatorial blue-footed booby population over the last ~100,000 years.

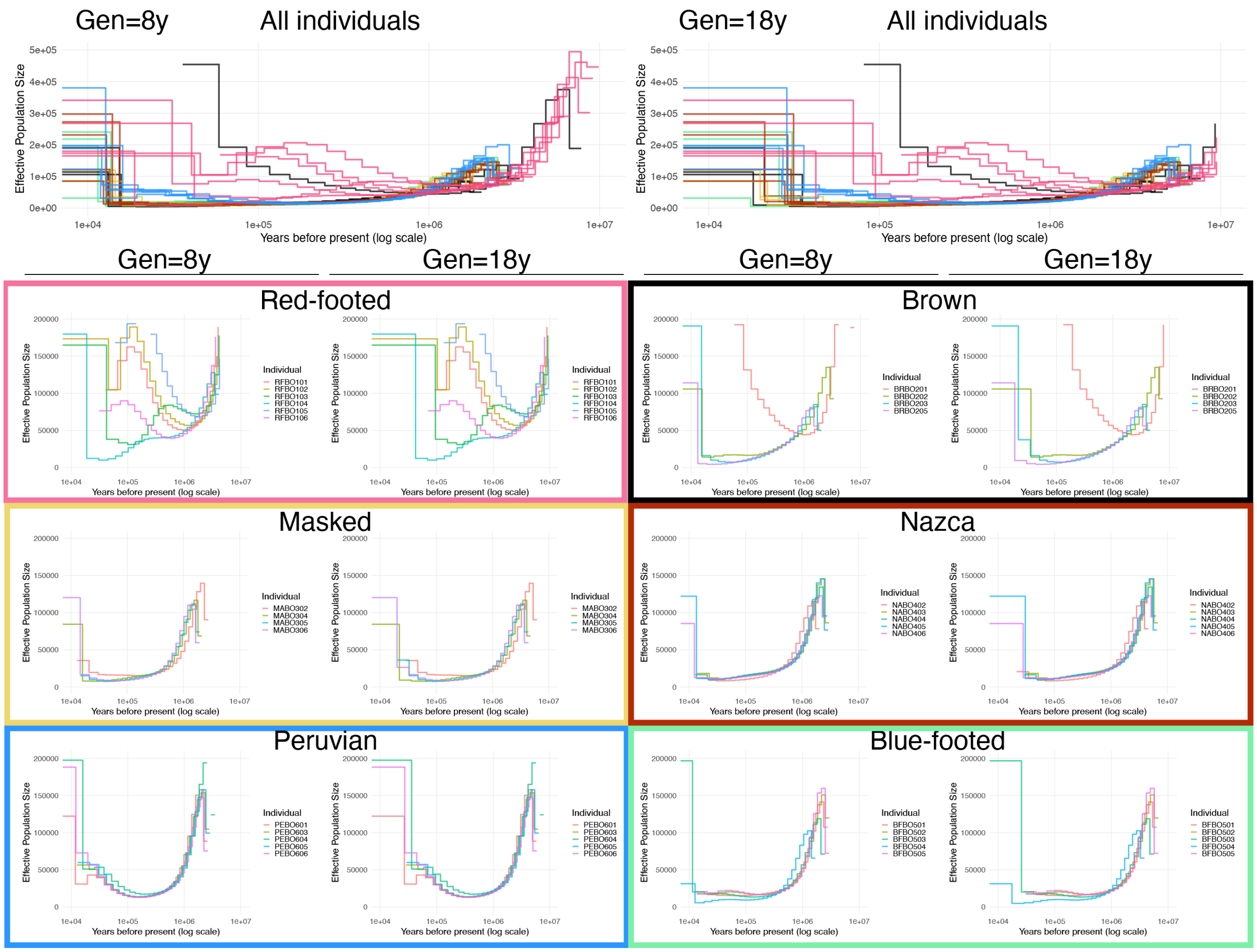

bioRxiv preprint doi: https://doi.org/10.64898/2026.07.17.739190; this version posted July 22, 2026. The copyright holder for this preprint (which was not certified by peer review) is the author/funder. This article is a US Government work. It is not subject to copyright under 17 USC 105 and is also made available for use under a CC0 license.

Figure S5: Semantic dissimilarity plots of enriched GO terms in outlier gene lists from interspecific F_{ST} comparisons.

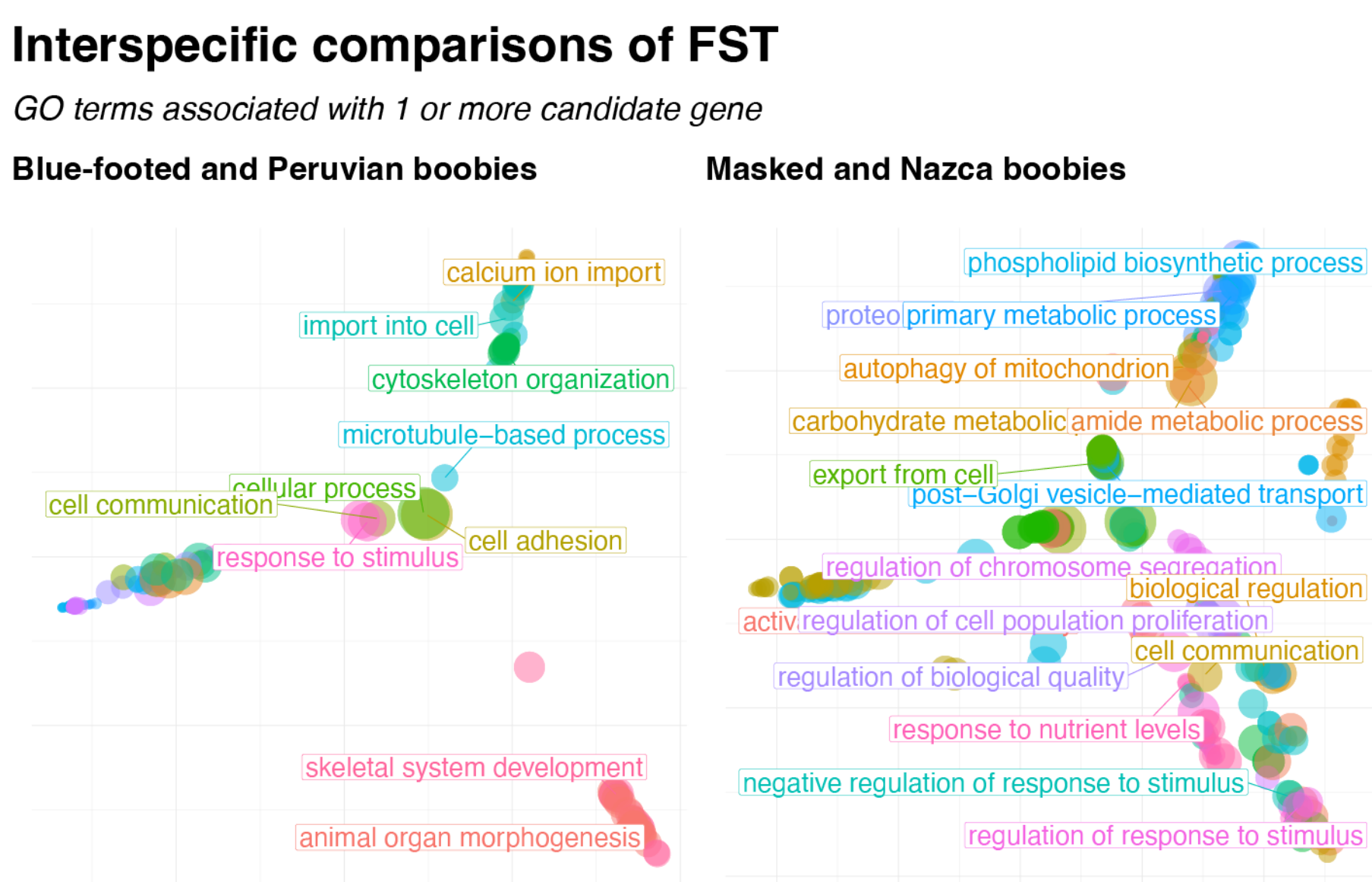

bioRxiv preprint doi: https://doi.org/10.64898/2026.07.17.739190; this version posted July 22, 2026. The copyright holder for this preprint (which was not certified by peer review) is the author/funder. This article is a US Government work. It is not subject to copyright under 17 USC 105 and is also made available for use under a CC0 license.

834 **Figure S6.** Heterozygosity across the neo-sex chromosome, faceted by species and colored by
835 individual.

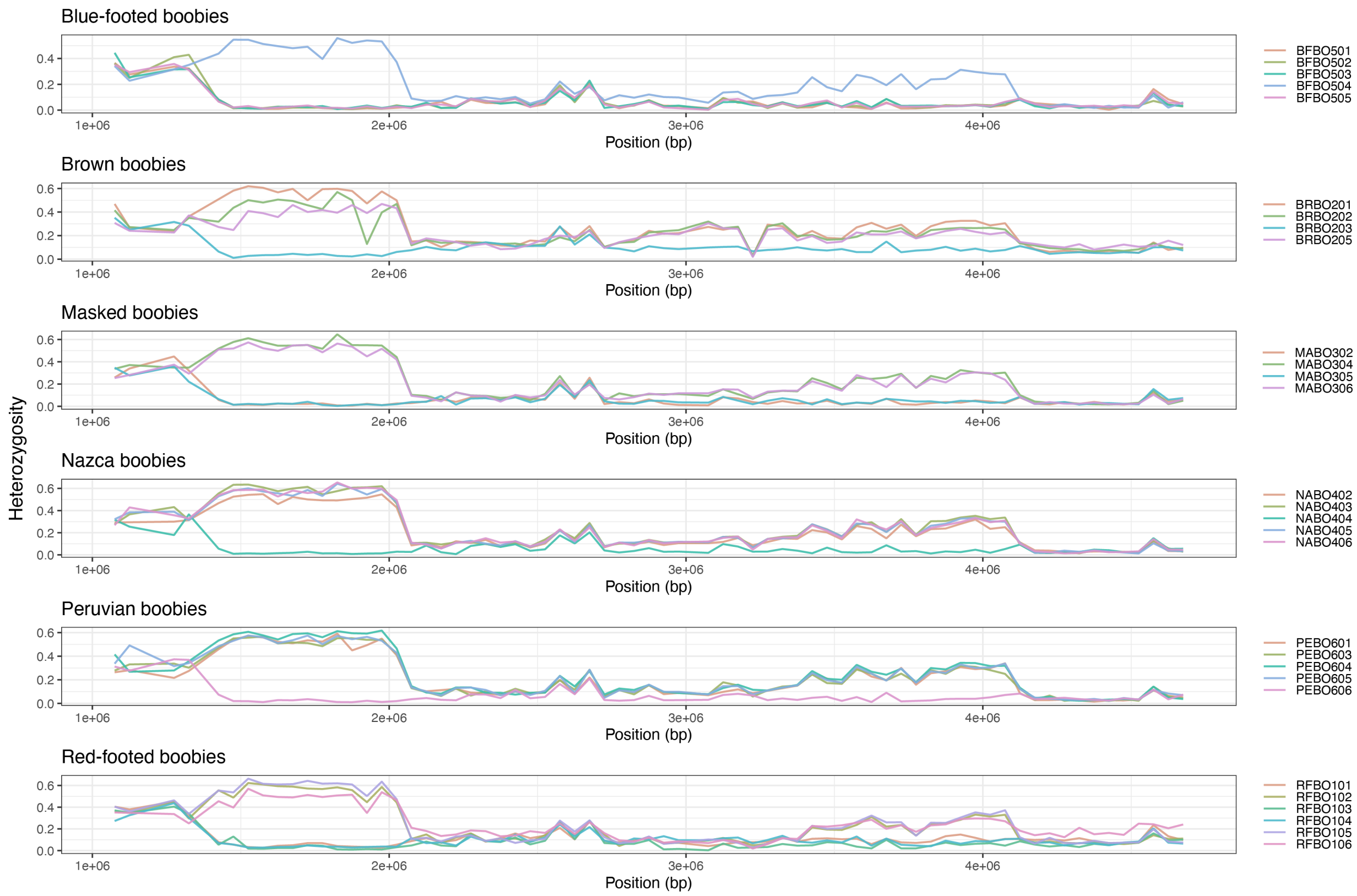

836

837

bioRxiv preprint doi: https://doi.org/10.64898/2026.07.17.739190; this version posted July 22, 2026. The copyright holder for this preprint (which was not certified by peer review) is the author/funder. This article is a US Government work. It is not subject to copyright under 17 USC 105 and is also made available for use under a CC0 license.

Figure S7: Semantic dissimilarity plot of enriched GO terms within region 2 of the neo-sex chromosome.

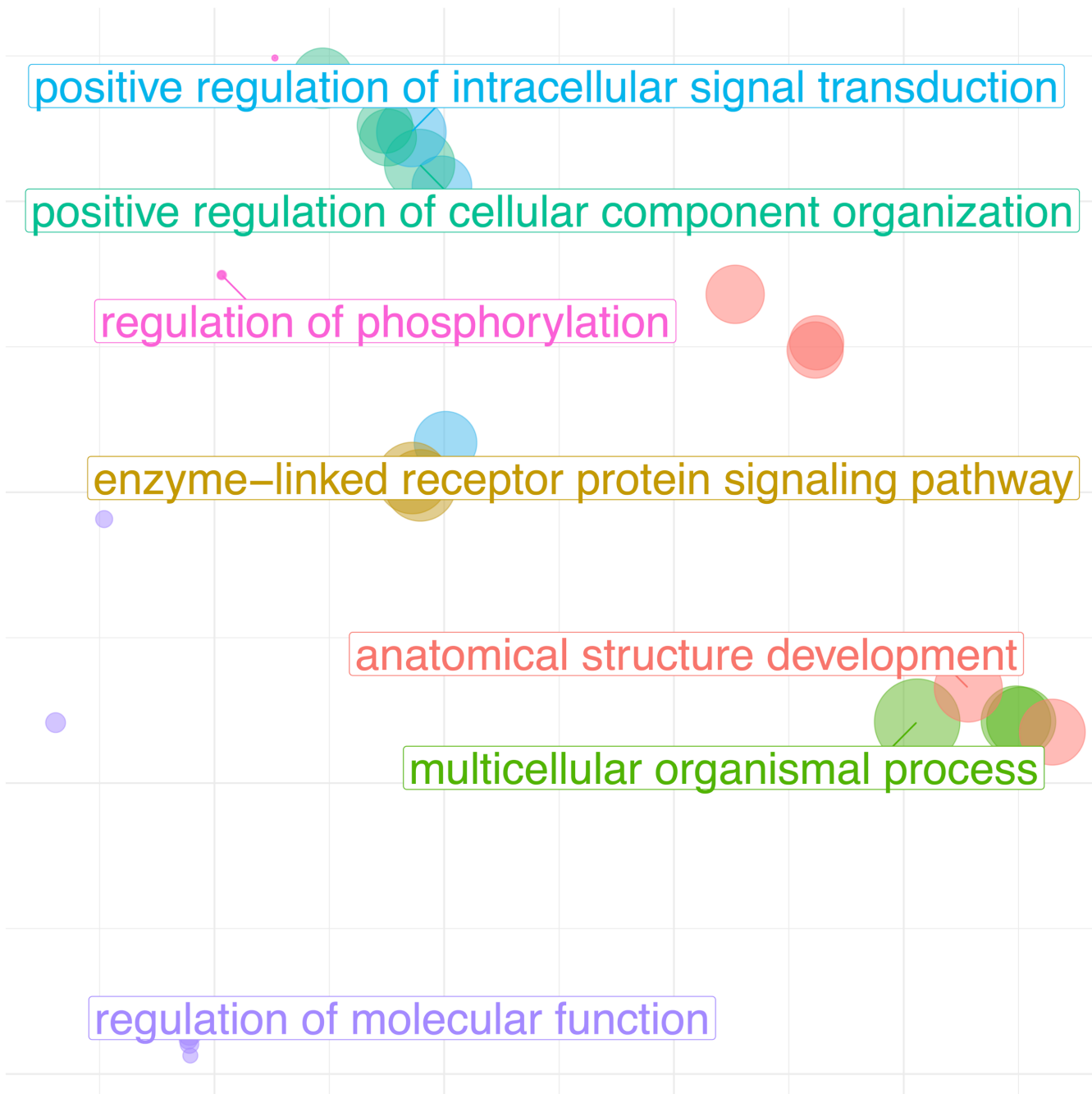

bioRxiv preprint doi: https://doi.org/10.64898/2026.07.17.739190; this version posted July 22, 2026. The copyright holder for this preprint (which was not certified by peer review) is the author/funder. This article is a US Government work. It is not subject to copyright under 17 USC 105 and is also made available for use under a CC0 license.

Figure S8: A. Depth of samples by chromosome and by inferred sex. The Z chromosome is the only chromosome with significant differences in depth between the sexes (Wilcoxon rank-sum test; Z chromosome: female mean H = XXX, male mean H = XXX, FDR corrected $p < 0.001$). **B.** Comparisons of heterozygosity and sequencing depth for inferring sex (using Z1) and inferring the identity of the neo-sex chromosome (Z2).

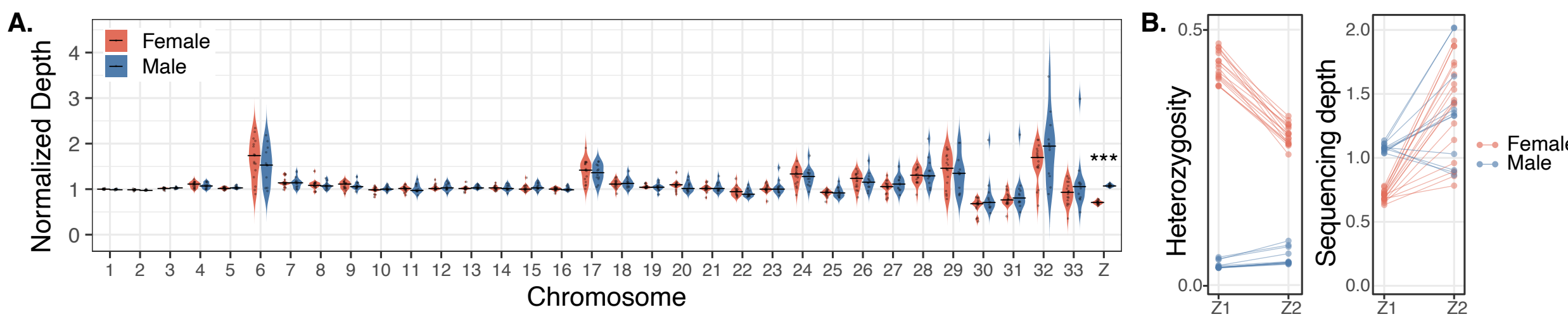

bioRxiv preprint doi: https://doi.org/10.64898/2026.07.17.739190; this version posted July 22, 2026. The copyright holder for this preprint (which was not certified by peer review) is the author/funder. This article is a US Government work. It is not subject to copyright under 17 USC 105 and is also made available for use under a CC0 license.

Supplemental Tables

Table S1. Depth of coverage. Depths were calculated from the final bam files, after all filtering efforts, but not including any downstream filtering steps made on the VCFs. Italicized individuals had an average depth below our cutoff of 3x and were excluded from all further analyses. We excluded blue-footed booby 6, brown booby 4, masked booby 1, Nazca booby 1, and Peruvian booby 2 from all analyses based on depth statistics. Values on the basis by which an individual was excluded from future analyses are highlighted in red.

Individual	Avg. Depth	SD Depth	Inferred sex
Blue footed 1	5.13	35.00	Male
Blue footed 2	5.39	31.24	Male
Blue footed 3	4.67	26.28	Male
Blue footed 4	3.90	27.92	Female
Blue footed 5	5.40	31.55	Male
Blue footed 6	**2.41**	42.58	NA
Brown 1	5.73	26.09	Female
Brown 2 (Cocos)	5.24	32.52	Female
Brown 3	4.01	28.16	Male
Brown 4	3.20	**162.60**	NA
Brown 5	3.73	37.39	Female
Masked 1	**2.90**	19.41	NA
Masked 2	5.43	18.77	Male
Masked 4	4.45	32.36	Female
Masked 5	4.13	32.67	Male
Masked 6	4.11	25.15	Female
Nazca 1	**2.58**	26.27	NA
Nazca 2	3.92	29.61	Female
Nazca 3	5.52	25.45	Female
Nazca 4	5.53	22.58	Male
Nazca 5	5.44	29.53	Female
Nazca 6	5.12	29.81	Female
Peruvian 1	4.15	18.06	Female
Peruvian 2	2.59	25.18	NA
Peruvian 3	4.41	36.39	Female
Peruvian 4	5.19	36.95	Female
Peruvian 5	4.38	35.38	Female
Peruvian 6	4.46	31.57	Male
Red footed 1	6.44	20.53	Male
Red footed 2	7.43	22.59	Female
Red footed 3	13.85	40.19	Male
Red footed 4	4.94	14.66	Male
Red footed 5	13.23	37.29	Female
Red footed 6	5.27	20.12	Female
Average	5.12	32.70	12 males
Avg. After exclusions	5.34	28.82	17 females

bioRxiv preprint doi: https://doi.org/10.64898/2026.07.17.739190; this version posted July 22, 2026. The copyright holder for this preprint (which was not certified by peer review) is the author/funder. This article is a US Government work. It is not subject to copyright under 17 USC 105 and is also made available for use under a CC0 license.

857 **Table S2: Significant tests of introgression using D-statistics with clustering-sensitive**
858 **and FDR corrected p-values.**

P1	P2	P3	D statistic	CS FDR q value	CR FDR q value
Gulf of CA blue footed	Equatorial blue footed	Atlantic/Caribbean brown	0.071	0.000	1.000
Gulf of CA blue footed	Equatorial blue footed	Pacific brown	0.046	0.000	1.000
Gulf of CA blue footed	Equatorial blue footed	Atlantic/Caribbean masked	0.150	0.000	1.000
Gulf of CA blue footed	Equatorial blue footed	Indopacific masked	0.153	0.000	1.000
Gulf of CA blue footed	Equatorial blue footed	Nazca	0.140	0.000	1.000
Gulf of CA blue footed	Equatorial blue footed	Peruvian	0.223	0.000	1.000
Pacific brown	Atlantic/Caribbean brown	Gulf of CA blue footed	0.250	0.000	1.000
Gulf of CA blue footed	Atlantic/Caribbean masked	Atlantic/Caribbean brown	0.039	0.008	1.000
Gulf of CA blue footed	Indopacific masked	Atlantic/Caribbean brown	0.044	0.007	1.000
Gulf of CA blue footed	Nazca	Atlantic/Caribbean brown	0.034	0.000	1.000
Gulf of CA blue footed	Peruvian	Atlantic/Caribbean brown	0.060	0.001	1.000
Gulf of CA blue footed	Atlantic/Caribbean masked	Pacific brown	0.030	0.004	1.000
Gulf of CA blue footed	Indopacific masked	Pacific brown	0.035	0.003	1.000
Gulf of CA blue footed	Nazca	Pacific brown	0.031	0.001	1.000
Gulf of CA blue footed	Peruvian	Pacific brown	0.054	0.003	1.000
Atlantic/Caribbean masked	Indopacific masked	Gulf of CA blue footed	0.013	0.001	1.000
Atlantic/Caribbean masked	Nazca	Gulf of CA blue footed	0.004	0.001	1.000
Gulf of CA blue footed	Peruvian	Atlantic/Caribbean masked	0.129	0.000	1.000
Nazca	Indopacific masked	Gulf of CA blue footed	0.007	0.002	1.000
Gulf of CA blue footed	Peruvian	Indopacific masked	0.130	0.000	1.000
Gulf of CA blue footed	Peruvian	Nazca	0.128	0.000	1.000
Pacific brown	Atlantic/Caribbean brown	Equatorial blue footed	0.269	0.000	1.000
Atlantic/Caribbean masked	Equatorial blue footed	Atlantic/Caribbean brown	0.012	0.000	1.000
Indopacific masked	Equatorial blue footed	Atlantic/Caribbean brown	0.006	0.000	1.000
Nazca	Equatorial blue footed	Atlantic/Caribbean brown	0.017	0.000	1.000
Peruvian	Equatorial blue footed	Atlantic/Caribbean brown	0.008	0.267	1.000
Atlantic/Caribbean masked	Equatorial blue footed	Pacific brown	0.002	0.001	1.000

bioRxiv preprint doi: https://doi.org/10.64898/2026.07.17.739190; this version posted July 22, 2026. The copyright holder for this preprint (which was not certified by peer review) is the author/funder. This article is a US Government work. It is not subject to copyright under 17 USC 105 and is also made available for use under a CC0 license.

Equatorial blue footed	Indopacific masked	Pacific brown	0.003	0.001	1.000
Nazca	Equatorial blue footed	Pacific brown	0.000	0.003	1.000
Equatorial blue footed	Peruvian	Pacific brown	0.014	0.131	1.000
Atlantic/Caribbean masked	Indopacific masked	Equatorial blue footed	0.016	0.000	1.000
Nazca	Atlantic/Caribbean masked	Equatorial blue footed	0.005	0.004	1.000
Peruvian	Equatorial blue footed	Atlantic/Caribbean masked	0.011	0.000	1.000
Nazca	Indopacific masked	Equatorial blue footed	0.020	0.018	1.000
Peruvian	Equatorial blue footed	Indopacific masked	0.014	0.001	1.000
Peruvian	Equatorial blue footed	Nazca	0.000	0.001	1.000
Pacific brown	Atlantic/Caribbean brown	Atlantic/Caribbean masked	0.259	0.000	1.000
Pacific brown	Atlantic/Caribbean brown	Indopacific masked	0.260	0.000	1.000
Pacific brown	Atlantic/Caribbean brown	Nazca	0.251	0.000	1.000
Pacific brown	Atlantic/Caribbean brown	Peruvian	0.254	0.000	1.000
Atlantic/Caribbean masked	Indopacific masked	Atlantic/Caribbean brown	0.008	0.013	1.000
Nazca	Atlantic/Caribbean masked	Atlantic/Caribbean brown	0.007	0.424	1.000
Atlantic/Caribbean masked	Peruvian	Atlantic/Caribbean brown	0.006	0.000	1.000
Nazca	Indopacific masked	Atlantic/Caribbean brown	0.015	0.133	1.000
Indopacific masked	Peruvian	Atlantic/Caribbean brown	0.001	0.000	1.000
Nazca	Peruvian	Atlantic/Caribbean brown	0.011	0.000	1.000
Atlantic/Caribbean masked	Indopacific masked	Pacific brown	0.007	0.005	1.000
Atlantic/Caribbean masked	Nazca	Pacific brown	0.002	0.051	1.000
Atlantic/Caribbean masked	Peruvian	Pacific brown	0.012	0.005	1.000
Nazca	Indopacific masked	Pacific brown	0.005	0.070	1.000
Indopacific masked	Peruvian	Pacific brown	0.007	0.000	1.000
Nazca	Peruvian	Pacific brown	0.010	0.009	1.000
Atlantic/Caribbean masked	Indopacific masked	Nazca	0.136	0.000	0.013
Atlantic/Caribbean masked	Indopacific masked	Peruvian	0.014	0.000	1.000
Atlantic/Caribbean masked	Nazca	Peruvian	0.005	0.000	1.000
Nazca	Indopacific masked	Peruvian	0.007	0.008	1.000
